# Effect of Focused Bedside Ultrasonography in Hypotensive Patients on the Clinical Decision of Emergency Physicians

**DOI:** 10.1155/2017/6248687

**Published:** 2017-03-05

**Authors:** M. Ikbal Sasmaz, Faruk Gungor, Ramazan Guven, K. Can Akyol, Nalan Kozaci, Mustafa Kesapli

**Affiliations:** ^1^Department of Emergency Medicine, Servergazi State Hospital, Denizli, Turkey; ^2^Department of Emergency Medicine, Antalya Training and Research Hospital, Antalya, Turkey; ^3^Department of Emergency Medicine, Bitlis State Hospital, Bitlis, Turkey

## Abstract

We assessed the effect of focused point of care ultrasound (POCUS) used for critical nontraumatic hypotensive patients presenting to the emergency department of our hospital on the clinical decisions of the physicians and whether it led to the modification of the treatment modality. This prospective clinical study was conducted at the Emergency Department of Antalya Training and Research Hospital. Nontraumatic patients aged 18 and older who presented to our emergency department and whose systolic blood pressure was <100 mmHg or shock index (heart rate/systolic blood pressure) was >1 were included in the study. While the most probable preliminary diagnosis established by the physician before POCUS was consistent with the definitive diagnosis in 60.6% (*n* = 109) of 180 patients included in the study, it was consistent with the definitive diagnosis in 85.0% (*n* = 153) of the patients after POCUS (*p* < 0.001). POCUS performed for critical hypotensive patients presenting to the emergency department is an appropriate diagnostic tool that can be used to enable the physicians to make the accurate preliminary diagnosis and start the appropriate treatment in a short time.

## 1. Introduction

Shock is a condition of acute circulator failure that leads to decreased organ perfusion due to insufficient oxygen supply to the tissues [1]. The effects of oxygen insufficiency are initially reversible; however, they may quickly become irreversible. As shock progresses, it results in consecutive cell death, target organ damage, multiple organ failure, and death [2].

Today, emergency departments are the places where patients with poor overall condition and critical patients access healthcare services. Majority of these critical patients have shock and hypotension of unknown cause. Diagnostic procedures and treatment have to be performed simultaneously during the medical care of these critical patients at the emergency department. If the diagnostic tests to be used to evaluate these patients are cheap, fast, and applicable at bedside, this will facilitate the work of the clinicians who race against time. One of the diagnostic tests is the use of point of care ultrasound (POCUS) that has been used by emergency medicine specialists for decades especially for traumatic patients and recently applied to critical patients as well. POCUS is getting more important in emergency medicine practices since it is reliable, rapid, noninvasive, and applicable at bedside [3].

Mortality rates in shock patients vary depending on the cause. While mortality due to septic shock is 40%–60%, mortality due to cardiogenic shock ranges between 36% and 56% [4]. It is very important to establish the diagnosis accurately and start the treatment early before organ dysfunction develops in order to reduce the morbidity and mortality in patients with shock. The definitive diagnosis of shock can be established early at the emergency department only in 25% to 50% of the cases [5, 6]. The accuracy of the diagnosis rises up to 80% with the use of POCUS performed in emergency department protocol [6]. The most important goal for the clinician is to determine the etiology of the current condition and avoid any delays in the supportive therapy.

Several resuscitation protocols in which ultrasound is used at an early stage for the care of critical patients have been developed recently [7–13]. Each of these protocols contains the same core ultrasound components; on the other hand, the most important difference between these protocols is the order of procedure priority. The RUSH (Rapid Ultrasound in Shock) protocol is one of the most comprehensive and effective ultrasonographic examination protocols for early detection and treatment. The RUSH protocol is evaluated at 3 respective steps (the pump, the tank, and the pipes) [8].

In this study, our primary research question was “Does the addition of a POCUS protocol performed by emergency physicians change initial diagnosis or management for emergency department patients with shock and hypotension of unknown cause?”

## 2. Material and Method

This single-center clinical prospective study was conducted on patients older than 18 years of age who presented to the Emergency Department of Antalya Training and Research Hospital with nontraumatic shock signs from 1 October 2013 to 1 December 2014. The emergency department of the hospital admits >400000 patients in a year. Before the study was kicked off, approval was obtained from the Clinical Research Ethics Board of Antalya Training and Research Hospital. The specialists, residents, nurses, and triage staff assigned at the emergency department were informed about the study prior to the implementation.

**Figure 1:**
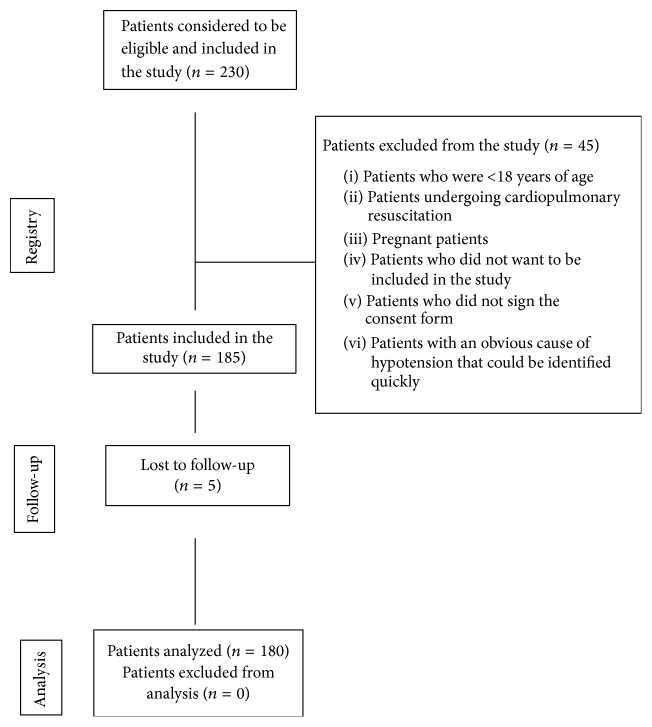
Patient flow chart.

Nontraumatic patients aged 18 and older who presented to the triage section of the emergency department and had a systolic blood pressure of <100 mmHg and/or shock index (pulse/systolic blood pressure) of >1 were given the study form and admitted to the suitable care point. The vital parameters of the patients taken to the suitable care point were reevaluated by the nurse. Those patients who met the inclusion criteria of the study were included in the study according to the results of this evaluation. This study was conducted by the emergency medicine specialists who had been working at the emergency department actively on 24-hour basis and received training on POCUS for the RUSH protocol. The concerned training is delivered by the faculty members who are entitled to issue certificates in this field. The emergency medicine specialists who are willing to participate in this training and complete it successfully are awarded a certificate. All of the emergency medicine specialists who conducted the study received the training. In Turkey, the curriculum of emergency medicine contains a module on POCUS. The training courses including also the RUSH protocol are delivered at large hospitals throughout the year on a regular basis. The training contained live models and simulators. We did not find any pathology at our hospital during the training. The demographic data, complaint at admission, the onset of the complaint, and vital parameters (arterial blood pressure, pulse, respiratory rate, fever, and pulse oximeter) as well as the preliminary diagnosis of the condition leading to hypotension after history-taking and physical examination were recorded in the study form. After the first part of the study form was filled out, the use of POCUS was performed for the patients according to the RUSH protocol by the same physician and the preliminary diagnosis after POCUS was recorded in the second part of the study form (Figure 1).

The definitive diagnosis of the patients who were hospitalized from the emergency department was established by 2 emergency physicians and 1 anesthesiologist in addition to the researcher in charge of the study according to their epicrisis reports. The patients who were discharged were advised to visit the outpatient clinic for follow-up while the definitive diagnosis of the patients who presented to the outpatient clinic was also established by these 3 outsider physicians based on the data recorded by the outpatient clinic. The patients who did not present to the outpatient clinic were excluded from the study. There was no disagreement between the outsider physicians with respect to the definitive diagnosis.

The exclusion criteria of the study were as follows: Trauma patients, pediatric patients aged <18, patients who underwent cardiopulmonary resuscitation, pregnant patients, patients who had acute myocardial infarct on ECG, patients who presented due to obvious causes (VT with pulse, external bleeding) and could be rapidly found to have hypotension (Figure 1).

The use of POCUS in the RUSH protocol was divided into six categories for evaluation.

### 2.1. Focused Cardiac Ultrasonography (FOCUS)

This ultrasonography is primarily used to evaluate the presence of pericardial effusion, volume of cardiac cavities, global cardiac function, and volume status of patients. The next step for those patients who are found to have pericardial effusion is to detect the right ventricular diastolic dysfunction for cardiac tamponade. The evaluation of left ventricular contractility is based on the estimation of changes in the ventricular volume during systole and diastole [14].

### 2.2. Inferior Vena Cava (IVC) Evaluation

The last guidelines of the American Society of Echocardiography support the use of IVC diameter and caval index to evaluate the volume status [15]. Caval index is used to evaluate the right atrial pressure in patients with spontaneous respiration [16]. Caval index is calculated with the following formulation: (*E*max − *I*max)/*E*max (*E*max: maximum diameter of IVC during expirium, *I*max: minimum diameter of IVC during inspirium).

IVC measurements are correlated with central venous pressure (CVP) and used to estimate CVP [17, 18]. A cut-off value of 2 cm for the maximal IVC diameter has a high sensitivity and specificity to predict right atrial pressure [19].

### 2.3. Focused Abdominal Ultrasonography

The right upper quadrant view was used to evaluate the presence of free liquid in the space between the liver and right kidney (Morison's pouch). Furthermore, the presence of free liquid in the right pleural space was also evaluated [20].

### 2.4. Evaluation of Abdominal Aorta

In order to rule out aneurysm, it is necessary to evaluate the entire abdominal aorta, especially paying attention to the area below the renal arteries where most of the abdominal aorta aneurysms (AAA) are located [21, 22].

### 2.5. Thoracic Ultrasonography

Thoracic ultrasonography can detect pulmonary edema characterized by the filling of lung parenchyma with liquid, which indicates volume overloading [23, 24]. In order to evaluate the presence of pulmonary edema through ultrasonography, patients' lungs were viewed with a low-frequency probe from the anterolateral region at the 2nd and 5th intercostal spaces [25].

### 2.6. Ultrasonography Imaging of Lower Limb Veins

If a thromboembolic event is suspected as the cause of shock, the next step must be to evaluate the lower limb veins. Most of the pulmonary embolism cases are caused by thrombosis in deep veins of lower limbs; therefore, limited compression of specific areas in the legs should be evaluated through ultrasonography. Compression ultrasound performed by applying direct pressure on the vein with a high-frequency linear probe has a high sensitivity of detecting DVT (deep vein thrombosis) [26, 27].

The ultrasonographic evaluation of the patients included in our study was performed with ESAOTE® MYLAB CLASS-C Color Doppler Ultrasound system that is routinely used for patient care at our emergency department. This ultrasound device contains a high-frequency LA 533/3.0–13.0 MHz linear probe, a CA 541/1.0–8.0 MHz convex probe, and a PA 240/1.0–4.0 MHz sector probe. The linear probe with a surface area of 22 mm has a scanning depth of 163 mm, while the sector probe with a surface diameter of 44 mm has a scanning depth of 362 mm. The convex probe has a surface area of 50 mm. The ultrasound device used in our study records the data automatically. However, there was no need to perform a secondary evaluation.

The data of the study were analyzed in SPSS 20.0 software. The numerical variables were expressed as mean ± standard deviation, while the categorical variables were expressed as percentage. To compare two dependent groups, McNemar test was used for frequency data. All hypotheses were developed in two-way and 0.05 was considered as alpha critical value.

## 3. Results

185 out of 230 patients enrolled in the study met the inclusion criteria. 5 of 185 patients included in the study (lost to follow-up) were excluded from the study. 104 (57,8%) of 180 patients whose data were evaluated were male while 76 (42,2%) were female. The mean age of the patients was 63,33 ± 18,08 (the youngest was 18, the oldest was 100).

The most common reason why the patients included in the study presented to the emergency department was shortness of breath with 23.3% (*n* = 42). The complaints of the patients on admission to the emergency department are shown in Table 1. As regards the vital signs of the patients included in the study, the average systolic blood pressure was 82.05 ± 9.56, average diastolic blood pressure was 52.21 ± 9.49 mmHg, average count of pulse per minute was 105.40 ± 19.38, average respiratory rate per minute was 18.92 ± 6.40, and average temperature was 36.78 ± 0.

The results of focused POCUS assessment performed for shock patients with hypotension and shock index above 1 are shown in Table 2. The patients were assessed under four categories: focused cardiac assessment, focused abdominal and pleural assessment, abdominal aorta assessment, and assessment of lower limb veins.

The most suspected preliminary diagnosis prior to POCUS was sepsis with 23.9% (*n* = 43). While the preliminary diagnosis of sepsis remained unchanged in 17.3% of the patients (*n* = 31) after POCUS, the preliminary diagnosis was modified in 6.7% of the patients (*n* = 12). The changes in the most probable preliminary diagnosis of the patients included in the study after POCUS are presented in Table 3. Myocardial ischemia was the condition for which the preliminary diagnosis was modified at the highest level after POCUS.

**Figure 2:**
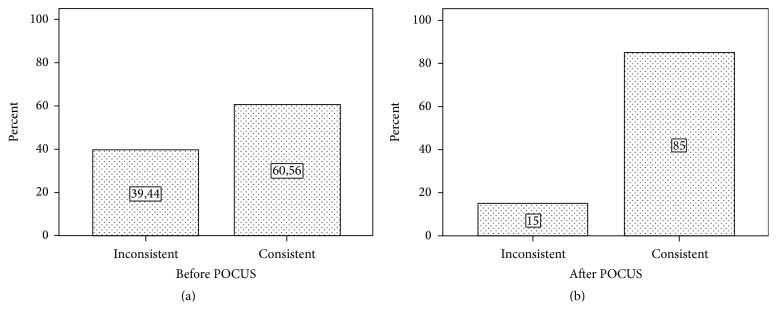
Consistency of preliminary diagnosis before and after POCUS with definitive diagnosis.

The preliminary diagnosis established by the physician prior to the use of POCUS was consistent with the definitive diagnosis in 60.6% of the patients (*n* = 109) included in the study. The rate of consistency between the preliminary diagnosis and definitive diagnosis after POCUS was 85.0% (*n* = 153). The preliminary diagnosis was modified in 32.2% (*n* = 58) of 180 patients after the use of POCUS. The consistency between the preliminary diagnoses before and after POCUS and definitive diagnoses is shown in Table 4 (*p* < 0.001) (Figure 2).

After the use of POCUS, the treatment plan was modified for 90 (50%) patients while a new treatment plan was developed for 40 (22,3%) patients after the use of POCUS. Similarly, the treatment plan developed for 50 (27,7%) before the use of POCUS was abandoned.

Based on the diagnosis and treatment results of the patients at the emergency department, 38.3% of the patients (*n* = 69) were discharged, 39.4% (*n* = 71) were taken to the intensive care unit, 16.1% (*n* = 29) were taken to the inpatient clinic, and 4.4% (*n* = 8) were referred while 1,7% (*n* = 3) were exitus at the emergency room.

The most common diagnosis of the patients included in the study was sepsis with 22.2% (*n* = 40) according to the assessment at the emergency department and definitive diagnosis established on the basis of the epicrisis reports of the inpatients. It was found that 16.7% of the patients (*n* = 30) were diagnosed with severe dehydration, 8.9% (*n* = 16) with left ventricular insufficiency, and 7,8% (*n* = 14) with vasovagal syncope. All the other diagnoses of the patients are shown in Table 5.

## 4. Discussion

Emergency departments are now intensively used by an increasing number of critical patients to access healthcare services due to the increased life expectancy and increased comorbidities. Hypotension of unknown cause and/or shock represent the majority of the critical patients admitted to the emergency department.

Hypotension detected at emergency department is one of the important markers of mortality in the hospital [28]. Therefore, it is vitally important to perform the diagnostic procedures and provide treatment simultaneously for the emergency medical care of patients with hypotension of unknown cause. If the diagnostic tests to be used to evaluate these patients are cheap, fast, and applicable at bedside, this will facilitate the work of the clinicians who race against time [3]. As in every area of medicine, POCUS has been increasingly used also in emergency medicine and is now an indispensable part of patient care. In emergency department, POCUS has allowed early diagnosis or exclusion of many important conditions in addition to enabling safer interventions, saving the lives of many patients, or enhancing the quality of care [13].

The application of POCUS in patients with hypotension of unknown cause at emergency department was first assessed in 2001 by Rose et al. in a series of 3 cases [9]. In the light of their findings in these case series, Rose et al. proposed a POCUS protocol for 3 sonographic areas (cardiac, Morison's pouch, and abdominal aorta). However, the disadvantage of this protocol is that it does not include the sonographic assessment of inferior vena cava that provides important information about the intravascular volume. Furthermore, the assessment of only Morison's pouch for the presence of free fluid in the abdomen does not seem to be adequate. The use of POCUS for hypotension of unknown cause and other critical clinical conditions was explored in many reviews and case series in the following years [7, 8, 10]. The two most widely used protocols are “Abdominal and Cardiac Evaluation with Sonography in Shock (ACES)” proposed in 2009 by Atkinson et al. [7] and “Rapid Ultrasound in Shock (RUSH)” proposed in 2010 by Perera et al. [8]. The purpose of these protocols was to assess the sonographic areas in a systematic way in order to accelerate the diagnosis process and start the appropriate treatment quickly. In ACES protocol, patients were assessed in 6 sonographic quadrants that included cardiac, pleural, peritoneal, IVC, and aortic quadrants. In RUSH protocol, however, thorax and lower limb veins were also added to the sonographic assessment in addition to the abovementioned quadrants. Assessment of pulmonary edema and pneumothorax with the use of thoracic POCUS in RUSH protocol appears to be an advantage compared to ACES protocol. Both protocols have been demonstrated to help the clinical assessment of patients presenting to the emergency department due to hypotension of unknown cause.

The effectiveness of POCUS in patients with hypotension of unknown cause was first explored in 2004 by Jones et al. in a prospective study [5]. The study showed that the use of POCUS for patients with hypotension of unknown cause decreased the preliminary diagnoses of the clinician and helped the diagnoses. In that study, they also assessed the left upper quadrants. In a study conducted in 2012 by Haydar et al. on patients diagnosed with septic shock, cardiac assessment and VCI measurements were used in their focused ultrasonography protocol [29]. They modified the treatment plan after the use of POCUS. Similarly in a study conducted in 2013 by Volpicelli et al. in Italy, they demonstrated that the use of multiple organ POCUS assessments in a focused protocol for patients with hypotension of unknown cause had a positive impact on the clinical decision and the initial treatment approaches [30].

**Figure 3:**
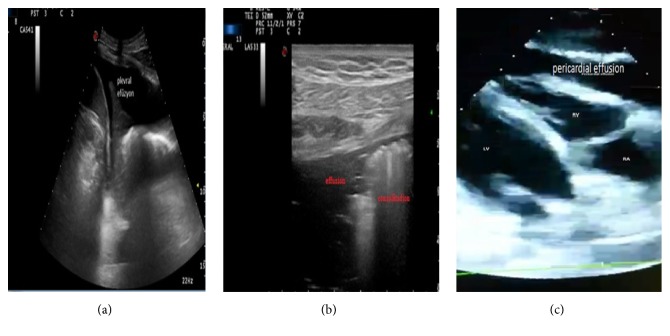
Some of the ultrasonographic pathologic views obtained during the study. (a) Pleural effusion view in the left pleural area in the left upper quadrant ultrasonography. (b) Pneumonic consolidation and parapneumonic effusion view in thoracic ultrasonography. (c) View of pericardial effusion compressing the left ventricle on the parasternal scan in the cardiac ultrasound.

Similarly to the study conducted in 2004 by Jones et al. and the study conducted in 2013 by Volpicelli et al., we also found in our study that the use of POCUS helped the diagnostic decisions of the physician significantly, increased the accuracy of diagnosis, and decreased the rate of misdiagnosis [5, 30]. Ghane et al. found in their study in 2015 that the RUSH protocol applied to shock patients was effective in typing and ruling out shock like we found in our study [31]. Moreover, the POCUS was found to have an important impact on the treatments provided for especially fatal diseases diagnosed in our study. Life-saving treatment was applied to 6 patients (thromboembolism therapy for 3 patients, pericardiocentesis for 3 patients) following POCUS at the emergency department without a need for other diagnostic tools. On the other hand, 7 patients were operated on urgently following POCUS (4 patients with hemoperitoneum/retroperitoneal bleeding, 3 patients with AAA rupture) (Figure 3).

## 5. Limitations

Our study was conducted in a single center, which might limit the generalizability of our results. The disadvantage of the study is that the sonographic findings of POCUS are quite similar in distributive shock and hypovolemic shock. In both conditions, left ventricular contractility and caval index are expected to increase. Although the thoracic assessment with POCUS may reveal findings that are consistent with pneumonia in this group of patients and this may make one suspect that the cause of shock is associated with sepsis, its differential diagnosis capability is low [32]. Despite that, aggressive fluid therapy that should be provided in both shock conditions can be started safely and rapidly following the use of POCUS. Another limitation of the study is that POCUS is dependent on the operator and the training/skill level of the clinicians.

## 6. Conclusion

The use of focused bedside POCUS increases the accuracy of diagnosis of hypotensive critical patients at the emergency department and improves treatment plans.

## Figures and Tables

**Table 1:** Complaints of patients on admission to the emergency department.

Complaints on admission	Number	%
Shortness of breath	42	23,3
Syncope	19	10,6
Abdominal pain	18	10,0
Poor overall condition	18	10,0
Near-syncope, feeling faint, blackout	16	8,9
Malaise	12	6,7
Oral intake disorder	7	3,9
Bloody stool/vomiting	7	3,9
Fever	6	3,3
Chest pain	6	3,3
Impaired consciousness	5	2,8
Palpitation	4	2,2
Other	20	11,1

**Table 2:** Findings of POCUS applied to shock patients.

Focused cardiac assessment	Present	
	*N*	%
Pericardial effusion	17	9,4
Diastolic pressure in right spaces	3	1,1

*Left ventricular contractility*	Incidence *n*:	

Hyperdynamic	70	
Normal-slightly decreased	83	
Decreased	27	

*Fractioned shortening*	Incidence *n*:	

<%30	27	
%30–%45	71	
>%45	82	

	Present	
	*N*:	%

Right ventricular hypertrophy	11	6,1
Septal displacement	4	2,2
Dilated aortic root	5	2,7
Intimal flap	0	0

	Present	
	*N*:	%

Vena cava collapse (caval index > %50)	78	43,4

Focused abdominal and pleural assessment	Present	
	*N*:	**%**

Hepatorenal fluid	12	6,7
Right pleural effusion	20	11,1
Splenorenal fluid	2	1,1
Left pleural effusion	17	9,4

**Table 3:** Changes in preliminary diagnosis after POCUS.

Preliminary diagnosis before USG	Total *N* (%)	No change in preliminary diagnosis after USG *N* (%)	Change in preliminary diagnosis after USG *N* (%)
Sepsis	43	31	12 (27.9)
Severe dehydration	34	26	8 (23.5)
Myocardial ischemia	9	1	8 (88.8)
GIS bleeding	10	8	2 (20.0)
Intra-abdominal infection	10	5	5 (50.0)
Left ventricular insufficiency	16	13	3 (18.7)
Pulmonary embolism	5	3	2 (40.0)
Vasovagal syncope	9	7	2 (22.2)
COPD acute attack	4	2	2 (50.0)
Other	39	26	13 (33.3)
Total	180 (100)	122 (67.8)	58 (32.2)

**Table 4:** Comparison of the preliminary diagnosis before and after USG and definitive diagnosis of patients included in the study.

	Preliminary diagnosis before USG		Preliminary diagnosis after USG		*p* value
	*N*	%	*N*	%	
Consistent with definitive diagnosis	109	60,6	153	85	<0.001
Inconsistent with definitive diagnosis	71	39,4	27	15	
Total	180	180	180	100	
Measure of agreement Kappa	Kappa index = 0.564 Moderate agreement		Kappa index = 0.820 Almost perfect agreement		

**Table 5:** Definitive diagnosis of patients included in the study.

	Incidence	%
Sepsis	40	22,2
Severe dehydration	30	16,7
Myocardial ischemia	3	1,7
Left ventricular insufficiency	16	8,9
Vasovagal syncope	14	7,8
Intraabdominal infection	8	4,4
Dysrhythmia	7	3,9
GIS bleeding	6	3,3
Pulmonary thromboembolism	5	2,8
Anemia	5	2,8
Hemoperitoneum/retroperitoneal hematoma	4	2,2
Cor pulmonale	4	2,2
Acute renal failure	3	1,7
Drug side effect	3	1,7
Cardiac tamponade	3	1,7
Massive pleural effusion	3	1,7
Rupture of Abdominal Aortic aneurism	3	1,7
Other	23	12,7
